# Influence of Pyranose and Spacer Arm Structures on Phloem Mobility and Insecticidal Activity of New Tralopyril Derivatives

**DOI:** 10.3390/molecules22071058

**Published:** 2017-06-25

**Authors:** Yao Chen, Zhi Wei Lei, Ying Zhang, Wen Yang, Hui Fang Liu, Yu Feng Zhou, Mao Fa Yang

**Affiliations:** 1 Institute of Entomology, Guizhou University, Guiyang 550025, Guizhou, China; cheny0221@163.com; 2 Guizhou Provincial Key Laboratory for Agricultural Pest Management of the Mountainous Region, Guizhou University, Guiyang 550025, Guizhou, China; 3 Guizhou Tea Research Institute, Guizhou Academy of Agricultural Sciences, Guiyang 550006, Guizhou, China; leizhiwei816@163.com (Z.W.L.); yangwen3409@126.com (W.Y.); 18300865026@163.com (H.F.L.); gzzhouyf@163.com (Y.F.Z.); 4 Guizhou Institute of Plant Protection, Guizhou Academy of Agricultural Sciences, Guiyang 550006, Guizhou, China; zhangying201201@126.com

**Keywords:** glucuronic acid, glucose, tralopyril, chlorfenapyr, phloem mobility

## Abstract

Six new conjugates were designed and synthesized by introducing glucose, methyl glucuronate or glucuronic acid moieties on tralopyril. Phytotoxicity and phloem mobility results demonstrated that the introduction of glucose, methyl glucuronate or glucuronic acid moieties can simultaneously solve the tough phytotoxicity problem and phloem mobility transformation of tralopyril. Conjugates **12** and **18** containing the glucuronic acid moiety exhibited higher phloem mobility than conjugates **9**, **11**, **15** and **17**. Conjugates **15**, **17** and **18** with methoxymethyl groups on the tralopyril pyrrole nitrogen atom showed activity against *Plutella xylostella*, while conjugates **9**, **11** and **12** with a methene group on the pyrrole N showed no activity. Cabbage roots were incubated in a buffered solution containing conjugates **15**, **17** and **18** at 4 mM for 72 h. Only **18** showed systemic insecticidal activity with 100% mortalityagainst *P. xylostella,* while **15** and **17** showed lower activity andchlorfenapyr showed no activity. The glucuronic acid promoiety imparted more phloem mobility to tralopyril than glucose and methyl glucuronate. The methoxymethyl group bond on the tralopyril skeleton was the key factor in determining the insecticidal activity of the conjugates. A promising systemic proinsecticide containing glucuronic acid and tralopyril moieties was proposed.

## 1. Introduction

For various practical reasons insecticides with phloem mobility are preferred for pest species hidden on unpredictable non-exposed plant parts, such as growing tips, roots, inside leaf deformations, and galls. However, there are very few existing synthetic insecticides with phloem mobility. Currently, only spirotetramat can be bidirectionally transported in plants [1]. Attempts have been made to achieve phloem-mobile insecticides by introducing a carboxyl group [2,3], amino acid [3], or sugar [4,5] into the parent compounds of existing non-phloem-mobile types. Three research groups have focused on the addition of a sugar moiety [4,5,6,7,8,9].

In 1995, Hsu et al. described a phloem-mobile pronematicide to solve the inherent incompatibility between phloem mobility transformation and activity loss by structural modification of pesticides [4]. Two conjugates, hydroxymethyloxamyl glucuronide (JR522) and its methyl ester (JM775), were successfully synthesized. These conjugates showed more phloem-mobile attributes and greater nematicidal activity than oxamyl alone. However, the applied strict reaction conditions limited further development of hydroxymethyloxamyl glucuronides.

Since 2011, Xu and his team have made great efforts to improve the phloem mobility of pesticides through the addition of a sugar group. Yang et al. described the phloem mobility of a conjugate containing glucose and fipronil (GTF), and they reported that GTF can be reconverted into fipronil in adult castor bean plants [5]. Subsequent studies on the phloem transport mechanism of GTF proved that the uptake and phloem transport of GTF involved a plant sugar carrier system [6]. Yuan et al. synthesized 10 conjugates containing both fipronil and monosaccharide moieties through an *O*-glycosidic linkage; phloem mobility test results indicated that eight conjugates show phloem mobility [7]. Lei et al. confirmed that glucose conjugation at the C-2, C-3, C-4, and C-6 positions has a significant influence on the phloem mobility of GTF conjugates [8]. Qin et al. synthesized six conjugates containing both rotenone or dalpanol and monosaccharide moieties; their phloem systemicity tests showed that only d-glucose conjugates exhibit phloem transport properties, and phloem mobility is affected by the parent molecule [9]. The above mentioned studies compared the differences in the phloem mobility among different monosaccharide moieties, and the effect of glucose positions on phloem mobility. In 2016, Bonnemain and his team had demonstrated that glutamic acid imparts more phloem mobility to fenpiclonil than glucose [10]. However, differences in the phloem mobility of phloem-mobile derivatives among glucose, methyl glucuronate and glucuronic acid promoieties have not yet been reported.

**Figure 1:**
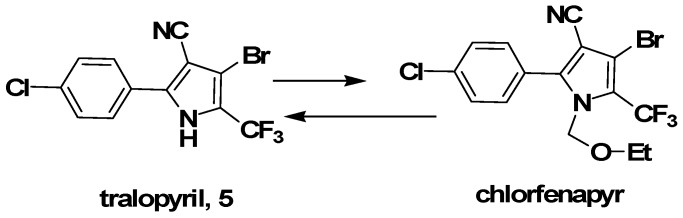
Chemical structure of tralopyril and chlorfenapyr.

Chlorfenapyr (Figure 1) is the first commercial pesticide derived from a class of compounds produced by bacteria and known as halogenated pyrroles [11]. This specific pesticide is a pro-insecticide with an ethoxymethyl group on the pyrrole N of tralopyril (Figure 1), which is the primary active compound [12,13]. Modification by ethoxymethyl group introduction is necessary to solve the intolerable phytotoxicity of tralopyril to the applied plants. Tralopyril uncouples oxidative phosphorylation in the mitochondria, resulting in the disruption of ATP production and loss of energy. These processes can lead to cell dysfunction and subsequent death of the organism. A recent study on phloem-mobile proinsecticides used tralopyril, which is a non-phloem-mobile candidate, to impart phloem mobility by introducing theanine or glutamic acid moieties [14]. Insecticidal activity test results indicated that amethoxymethyl group linker on thepyrrole N of the conjugates was dramatically more favorable to activity than a methene group. However, this structure–activity phenomenon has not yet been extended to other promoieties such as sugars.

The experimental scheme in the present study can be described as follows: (I) Six new tralopyril conjugates containing glucose, methyl glucuronate or glucuronic acid moieties were synthesized. (II) Their phytotoxicities to Fuding white tea seedlings were tested. (III) A phloem mobility test in *Ricinus communis* L. seedlings was performed. Their phloem mobility mechanism was predicted via the “Rule of Five” and a Kleier model. (IV) A poison fodder method was adopted to determine the insecticidal activity of the conjugates against the third-instar larvae of *Plutella xylostella*. (V) Their systemic mobility on cabbage and systemic insecticidal activity against the third-instar larvae of *P. xylostella* were evaluated. This study was performed to investigate the influence of different sugar promoieties on phloem mobility and insecticidal activity. Moreover, the influence of the two linkage arms (a methene and a methoxymethyl group) between the sugar promoiety and tralopyril on insecticidal activity was determined.

## 2. Results and Discussion

### 2.1. Synthesis and Characterization

**Scheme 1:**
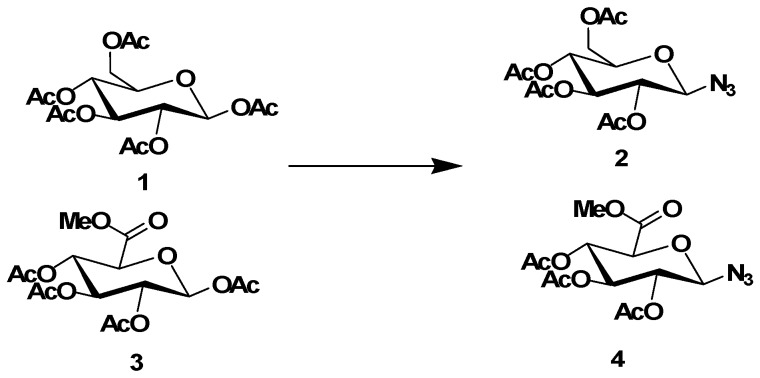
Synthesis of Azide Intermediates **2** and **4**. Reagents and conditions: azidotrimethylsilane, SnCl_4_, CH_2_Cl_2_, rt, 2 h.

**Scheme 2:**
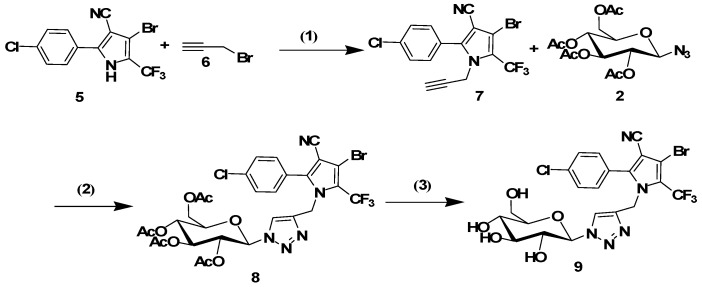
Synthesis of glucose conjugate **9** with a space arm of methene group. Reagents and conditions: (1) NaH, THF, 65 °C, 24 h; (2) CuSO_4_·5H_2_O (0.4 equiv), sodium ascorbate (0.8 equiv), 1:1 t-BuOH/H_2_O, 60 °C, 3 h; (3) 0.05 M NaOMe/MeOH, rt, 0.5 h, Amberlite IR 120H^+^.

**Scheme 3:**
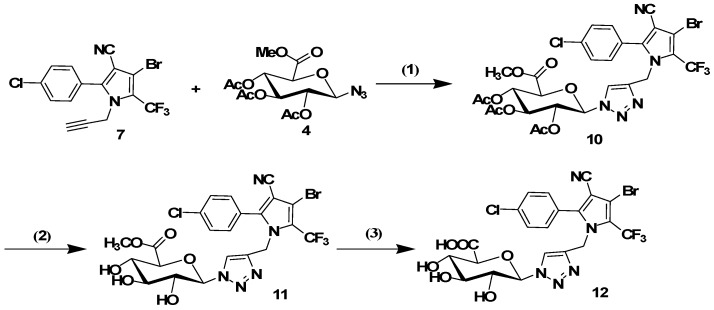
Synthesis of methyl glucuronate or glucuronic acid conjugate**s** with a space arm of methene group. Reagents and conditions: (1) CuSO_4_·5H_2_O (0.4 equiv), sodium ascorbate (0.8 equiv), 1:1 t-BuOH/H_2_O, 60 °C, 3 h; (2) 0.05 M NaOMe/MeOH, Room Temperature, 0.5 h, Amberlite IR 120H^+^; (3) KOH, MeOH, 0 °C, rt, overnight, Amberlite IR 120H^+^.

**Scheme 4:**
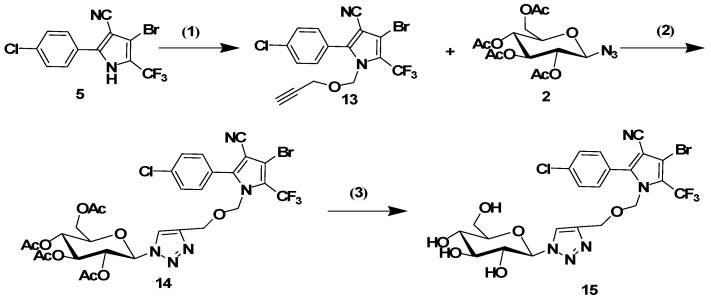
Synthesis of glucose conjugate **15** with a space arm of methoxymethyl group. Reagents and conditions: (1) THF, Bromochloromethane(10 equiv), NaH, Propargyl alcohol, 65 °C, 48 h; (2) CuSO_4_·5H_2_O (0.4 equiv), sodium ascorbate (0.8 equiv), 1:1 t-BuOH/H_2_O, 60 °C, 3 h; (3) 0.05 M NaOMe/MeOH, rt, 0.5 h, Amberlite IR 120H^+^.

**Scheme 5:**
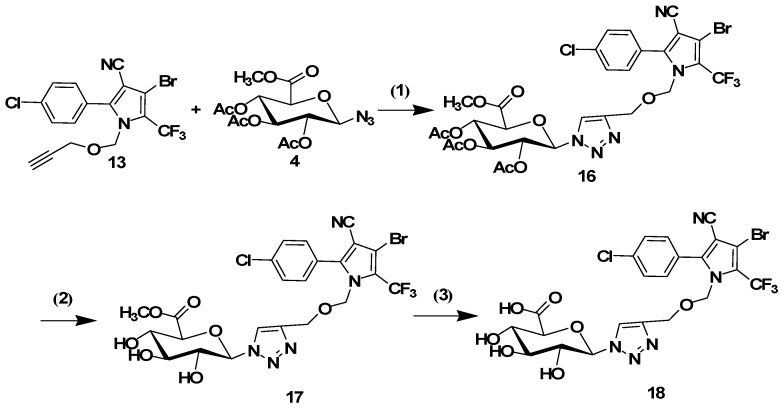
Synthesis of methyl glucuronate or glucuronic acid conjugate**s** with a space arm of methoxymethyl group. Reagents and conditions: (1) CuSO_4_·5H_2_O (0.4 equiv), sodium ascorbate (0.8 equiv), 1:1 t-BuOH/H_2_O, 60 °C, 3 h; (2) 0.05 M NaOMe/MeOH, 45 °C, 1 h, Amberlite IR 120H^+^; (3) KOH, MeOH, 0 °C, rt, over-night, Amberlite IR 120H^+^.

Conjugates that share structural similarities should be designed and synthesized in order to accurately compare the differences in phloem mobility impartedby glucose, methyl glucuronate and glucuronic acid moieties to tralopyril. Therefore, azides **2** and **4** (Scheme 1)**,** which showed small structuraldifferences at the C-6 positions of pyranose, were selected and prepared according to previously reported procedures [15,16]. A propargyl group was introduced into tralopyril to afford **7** (Scheme 2) and **13** (Scheme 4) as a coupling partner for the subsequent click reactions. Scheme 2, Scheme 3, Scheme 4 and Scheme 5 show that the terminalalkynes in **7** and **13** were reacted with azide intermediates **2** and **4**, respectively, to afford intermediates **8, 10, 14** and **16** with yields up to 90% via click reactions. The removal of acetyl groups was performed by the Zemplén procedure. The resulting crude products were purified by column chromatography to yield the deprotected conjugates **9**, **11**, **15** and **17** as white solids. Conjugates **11** and **17** were de-esterified in the presence of KOH in MeOH to afford conjugates **12** and **18** as white solids.

The structures of conjugates **8**–**12** and **14**–**18** were confirmed by ^1^H-, ^13^C-NMR and ESI-MS spectroscopy. The characteristic signals of the 1,2,3-triazole and acetyl groups on the sugar rings could be observed in the ^1^H- and ^13^C-NMR spectra of conjugates **8**, **10**, **14** and **16**. In the ^1^H-NMRspectra of these compounds, single peaks corresponding to the 1,2,3-triazole and acetyl groups were found at 8.25–8.31 and 1.76–2.03 ppm; in their ^13^C-NMRspectra, the characteristic peaks of acetyl groups were found at 20 and 169–170 ppm. The removal of acetyl groups from conjugates **8**, **10**, **14** and **16** was confirmed by the ^1^H-and ^13^C-NMR spectra of conjugates **9**, **11**, **12, 15**, **17** and **18**. Thecharacteristic peaks at 1.76–2.03 ppm in the ^1^H-NMR spectra, and the peaks at 20 and 169–170 ppm in the ^13^C-NMR spectra disappeared after deprotection. The removal of the methyl group from conjugates **11** and **17** was confirmed by the ^1^H and ^13^C-NMR spectra of conjugates **12** and **18**. The characteristic peaks at 3.66–3.74 ppm in ^1^H-NMR spectra and the peaks at 52–53 ppm in the ^13^C-NMR spectra disappeared after deprotection. The β-configuration of conjugates **9**, **11**, **12, 15**, **17** and **18** was determined from the ^1^H-NMR spectroscopic data, in which H-1 appeared as a doublet, δ 5.45–5.50 (*J* = 9.2–9.4 Hz) for conjugates **9** and **15** as well as δ 5.56–5.71 (*J* = 9.3 Hz) for conjugates **11**, **12**, **17** and **18**. The mass spectra of the products exhibited excellent correlation with the calculated molecular mass.

### 2.2. Phytotoxicity to Tea Shoots

Chlorfenapyr is a pro-insecticide optimized from tralopyril which exhibits intolerable phytotoxicity to the applied plants [17]. The tea shoots were selected for phytotoxicity assay, mainly because chlorfenapyr could be used to control tea infection pests and it has a lower European Union MRLs in tea [14]. As shown in Table 1, no phytotoxicity symptoms were observed up to 63 days after smearing conjugates **9**, **11**, **12, 15**, **17** and **18** (0.02, 0.2, and 2 mM, respectively; score: 0–5%; grade 1) under pot experimental conditions. By contrast, tralopyril showed obvious phytotoxicity symptoms at 0.2 mM (score: 61–70%; grade 7) and 2 mM (score: 71–80%; grade 8), but not at 0.02 mM (score: 0–5%; grade 1). These results indicated that the introduction of a sugar group may solve the difficult phytotoxicity problem of tralopyril.

### 2.3. Phloem Mobility

**Figure 2:**
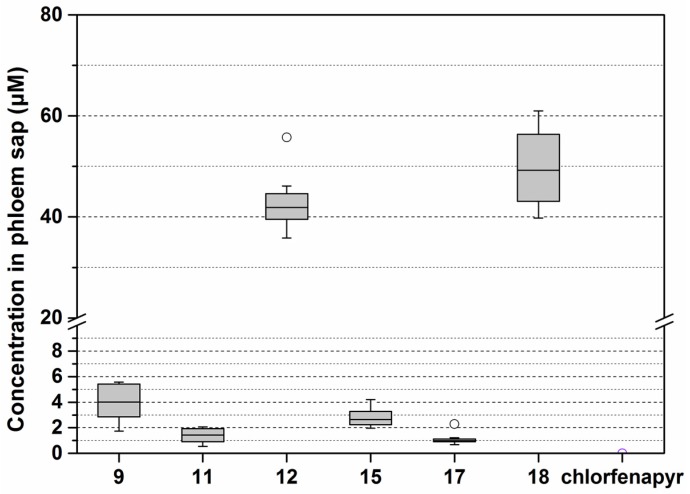
Phloem mobility of **9**, **11**, **12**, **15**, **17**, **18** and chlorfenapyr. The cotyledons were incubated in a buffered solution containing 200 μM **9**, **11**, **12**, **15**, **17**, **18** and chlorfenapyr at pH 5.5. The sap was collected at the third hour of incubation. For box plots, *n* = 8.

The castor bean system was selected for the phloem mobility assay, mainly because it is widely employed to evaluate phloem mobility of nutrients as well as xenobiotics [5]. The phloem sap of conjugates **9**, **11**, **12, 15**, **17**, **18** and chlorfenapyr was collected at 3 h for detection. When *Ricinus* cotyledons were incubated with 200 µM conjugates **9**, **11**, **12, 15**, **17** and **18** for 3 h, they were clearly detected, and their concentrations in the phloem sap were about 4, 1.5, 40, 2.5, 1 and 50 µM, respectively (Figure 2). When the cotyledons were incubated in chlorfenapyr solution at the same concentration, it was not detected in the phloem sap (Figure 1). 

These results indicated that the introduction of the three different sugar promoieties could impart phloem mobility to tralopyril. Based on the concentrations of conjugates **9**, **12**, **15** and **18** for 3 h in the phloem sap, the concentration of conjugates **12** and **18** in the phloem sap was approximately 10-fold and 20-fold greater higher than that of conjugates **9** and **15**, which showed that the glucuronic acid moiety caused higher phloem mobility than the glucose moiety. The concentration of conjugates **12** and **18** was approximately 27-fold and 50-fold greater than that of conjugates **11** and **17**. This result indicates that the carboxyl group has a positive contribution to phloem mobility. Conjugates **9** and **15** shared the same glucose and 1,2,3-triazole moieties with GTF, but their concentrations were approximately 0.1-fold greater than that of GTF at 3 h [5]. This result again demonstrates that phloem mobility was affected by the parent molecule.

**Figure 3:**
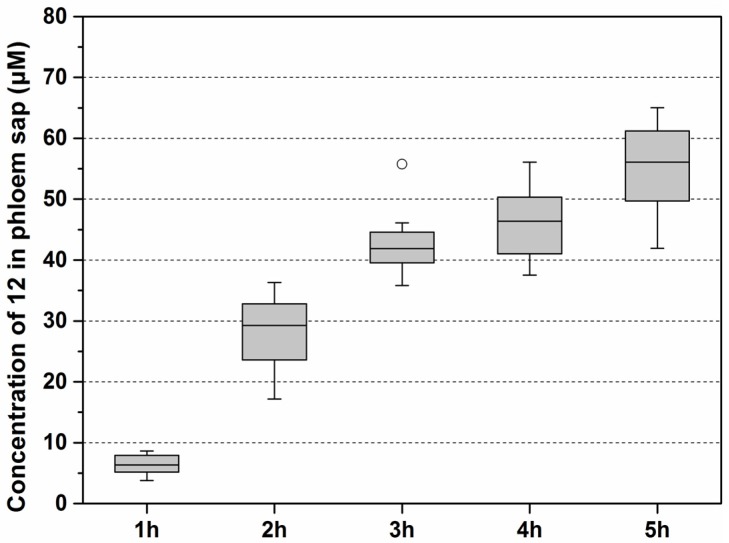
Time course of conjugate **12** concentration in phloem sap of R. communis. The cotyledons were incubated in a buffered solution containing 200 μM **12** at pH 5.5. The hook was severed at time 1 h, and then the sap was collected every 1 h during 5 h. For box plots, *n* = 8.

**Figure 4:**
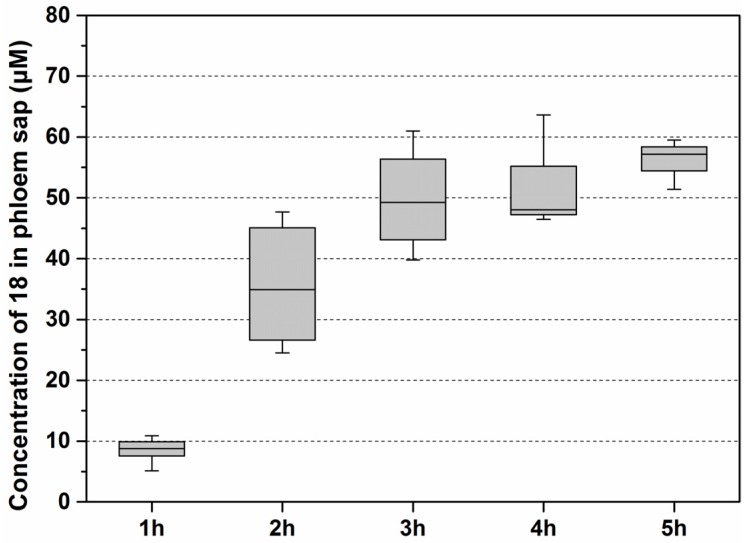
Time course of conjugate **18** concentration in phloem sap of R. communis. The cotyledons were incubated in a buffered solution containing 200 μM **18** at pH 5.5. The hook was severed at time 1 h, and then the sap was collected every 1 h during 5 h. For box plots, *n* = 8.

Time-course experiments were conducted during a 5 h period to further elucidate the time-course phloem mobility for conjugates **12** and **18**. The results (Figure 3 and Figure 4) indicated that the concentrations of conjugates **12** and **18** in the phloem sap increased during the test period. Their concentration reached 55 µM and 56 µM at 5 h, which was approximately 9-fold and 7-fold higher than those at 1 h.

The effectiveness of the addition of a carboxyl group, amino acid, or sugar to the parent compound for converting a non-mobile crop protection product into a phloem-mobile type was proven. In the first approach, uptake and translocation of conjugates may involve an ion-trapping effect. For example, the phloem mobility of acidic derivatives of the fungicide fenpiclonil was caused by the ion trap mechanism [2]. In the second approach, uptake and translocation of conjugates may involve two components of specific active carrier-mediated systems and passive transport. For example, 2,4D-Lys uptake involves an active carrier-mediated system aside from diffusion [20]. In the last approach, uptake and translocation of conjugates may involve a specific active carrier-mediated system. For example, the uptake and phloem transport of GTF involve an active carrier-mediated mechanism [6].

The “Rule of Five”, which is widely adopted in the pharmaceutical industry, can also be used to predict the diffusion of endogenous molecules or xenobiotics through plant membranes [21,22]. According to this rule, poor absorption or permeation of small molecules is more probable when their MW, log P, HBDs, and HBAs are more than 500D, 5, 5, and 10, respectively. Moreover, at least three in four parameters should satisfy these values [23]. Compound classes that are substrates for biological transporters are exceptions to this rule [23]. Considering that the two parameters (MW and HBAs) of conjugates **9**, **11**, **12, 15**, **17** and **18** in Table 2 are out of range, they were poorly diffused through the membranes. Therefore, a carrier system is probably involved in the long-distance transport of these conjugates. The Kleier model is also used to predict the phloem systemicity of a compound based on the physicochemical properties (log Kow pKa and log Cf) of molecules [24,25,26]. According to this model, large values of log Cf correspond to high predicted phloem mobility. As shown in Table 2, the introduction of a sugar moiety to the parent compound resulted in the lowering of log Kow for conjugates **9**, **11**, **12, 15**, **17** and **18**, but only the glucuronidation products **12** and **18** possessed lower pKa values. In terms of log Cf values, conjugates **9**, **11**, **15** and **17** were in the non-mobile molecule area, whereas conjugates **12** and **18** were in the mobile molecule area [2,6,25,26]. This observation partly explained why the phloem mobility of conjugates **12** and **18** were higher than that of conjugates **9**, **11**, **15** and **17**. As stated by Hsu et al., glucuronidation is indeed a good method to confer phloem mobility to immobile or poorly mobile pesticides. The effect of this process is exerted by a combined reduction in log Kow (over two units) and impartation of desirable acidity to exploit the ion-trapping effect [4]. Therefore, the uptake and translocation of conjugates **12** and **18** may involve two components of carrier-mediated systems and ion trap mechanism. This inference should be confirmed by obtaining direct evidence in further experiments.

### 2.4. Insecticidal Activity Against P. xylostella

The poison fodder method was adopted to determine the insecticidal activity of conjugates **9**, **11**, **12, 15**, **17** and **18** against *P. xylostella* at concentrations of 1, 2 and 4 mM. As shown in Table 3, when *P. xylostella* was exposed to 4 mM conjugates for 72 h**,** conjugates **9**, **11** and **12** with a methylene group on the pyrrole N of tralopyril moiety exhibited no activity, while conjugates **15**, **17** and **18** with a methoxymethyl group on the pyrrole N of tralopyril moiety showed 100% mortality. The results again proved that the methoxymethyl group on the pyrrole N of tralopyril moiety was akey factor to determine the insecticidal activity [14]. Among **15**, **17** and **18**, conjugate **15** with the glucose promoiety showed the lowest insecticidal activity, while **17** with the methyl glucuronate promoiety showed the best insecticidal activity. These observed differences indicated that the methyl glucuronate and glucuronic acid promoieties were clearly more favorable to the insecticidal activity to tralopyril than glucose. Therefore, further systemic insecticidal activity assessment of conjugates **15**, **17** and **18** were carried out.

### 2.5. Systemic Mobility on Cabbage and Systemic Insecticidal Activity

When 4 mM of conjugate **15** was root-applied, its detected concentrations after 48 h and 72 h were about 252 µg/g and 47 µg/g, respectively (Table 4). When 4 mM of conjugate **17** was root-applied, it was not detected, but conjugate **18** was detected (Table 4).This result is due to the fact that conjugate **17** was hydrolyzedin to conjugate **18** in aqueous solution. When 4 mM of conjugate **18** was root-applied, it was clearly detected, and its concentrations at 48 h and 72 h were about 1905 µg/g and 1947 µg/g, respectively (Table 4). By contrast, after root-application of 4 mM chlorfenapyr, it was not detected (Table 4). These observed differences indicate that the introduction of sugar promoieties could impart xylem mobility to tralopyril, and the glucuronic acid promoiety was clearly more favorable for the xylem mobility of tralopyril than glucose.

The harvested leaves from the treatments of root-applied conjugates **15**, **17** and **18** were used to determine the systemic insecticidal activity. Table 5 shows that when *P. xylostella* was exposed to the 48 h and 72 h harvested leaves, the treatments of root-applied conjugates **15**, **17** and **18** exhibited different insecticidal activities, especially conjugate **18** which showed excellent activity with a mortality of 93.33% and 100% at 48 h and 72 h. However, the harvested leaves from the treatments of root-applied chlorfenapyr showed no insecticidal activity. The results of the root-applied activity screening model proved that glucuronic acid promoiety imparts more systemic insecticidal activity to tralopyril than glucose and methyl glucuronate. 

Previous studies showed that specific promoiety introduction that confer phloem mobility to pesticides usually lower direct biological activity [3,4,5,14]. This phenomenon was also observed in the present work. Hsu et al. described the strategy of phloem-mobile pro-pesticide to overcome this inherent incompatibility [4]. Conjugate **18** may meet this strategy as the excellent systemic mobility and systemic insecticidal activity. Although, conjugate **18** exhibited lower direct insecticidal activity than chlorfenapyr, its excellent property of systemic insecticidal activity enables this conjugate to maintain the insecticidal activity for the insects hidden on unpredictable non-exposed plant parts.

## 3. Materials and Methods

### 3.1. Synthesis

#### 3.1.1. General Information 

All reagents were purchased from a commercial company. Analytical thin-layer chromatography (TLC) was performed on silica gel GF254 plates (Qingdao Marine Chemical Ltd., Qingdao, China). Silica gel (100–200 and 200–300 mesh, Qingdao Marine Chemical Ltd., Qingdao, China) was used for column chromatography. ^1^H-and ^13^C-NMR spectra were obtained on a WNMR-I 500 (Wuhan Institute of Physics and Mathematics (WIPM) of Chinese Academy of Sciences, Wuhan, Hubei, China) or INOVA 400 (Agilent Technologies Inc., Santa Clara, CA, USA,) instrument. Chemical shifts were expressed in δ (ppm) values, with TMS as an internal standard. The mass spectra of new conjugates were obtained by high-performance liquid chromatography (HPLC)-mass spectrometry (MS) (Thermo, San Jose, CA, USA) using electrospray ionization (ESI) and are reported as *m*/*z* values.

#### 3.1.2. Synthesis of Azide Intermediates **2** and **4**

Azide intermediates were synthesized according to previously reported procedures [15,16]. Briefly, compounds **1** or **3** (2 mmol) were added to a solution of azidotrimethylsilane (2.2 mmol) in anhydrous CH_2_Cl_2_ (10 mL), followed by dropwise addition of SnCl_4_ (2 mmol). The resulting mixture was stirred at room temperature (rt) for 2 h, then extracted three times with aqueous sodium hydrogen carbonate and washed with brine, dried over sodium sulfate, filtered, and evaporated in vacuo. The residues were then purified byrecrystallization from ethanol to give the azide intermediates **2** or **4** as white solids.

#### 3.1.3. Synthesis of *N*-Propargyl Tralopyril (**7**)

Synthesis of the title compound was accomplished according to a previously reported procedure [14]. Briefly, NaH (60% in oil, 0.144 g, 3.6 mmol) was added to the solution of tralopyril (1.05 g, 3 mmol) in THF (15 mL). The mixture was then stirred at room temperature for 2 h. Propargyl bromide (3.3 mmol) in THF (15 mL) was dropwise added at room temperature. The mixture was then stirred at 65 °C for 48 h. The reaction mixture was quenched by adding ice-water, and the resulting mixture was extracted with ethyl acetate (15 mL × 3). The combined organic layers were washed with aqueous sodium hydrogen carbonate and brine, dried with sodium sulfate, filtered, and evaporated in vacuo. The residue was purified by column chromatography (10 mL min^−1^) to afford the desired product **7**.

#### 3.1.4. Synthesis of *N*-Propynoxymethyl Tralopyril (**13**)

The title compound was synthesized according to a previously reported procedure [14]. Briefly, NaH (60% in oil, 0.288 g, 7.2 mmol) was added to the solution of tralopyril (1.05 g, 3 mmol) in mixture solvent of THF (20 mL) and bromochloromethane (1.95 g, 15 mmol, 5 equiv). The mixture was then stirred at room temperature for 2 h. Propargyl alcohol (3 mmol) in THF (10 mL) was dropwise added at 65 °C. The mixture was then stirred at 65 °C for 48 h. The reaction mixture was quenched by adding ice-water, and the resultant mixture was extracted with ethyl acetate (20 mL × 3). The combined organic layers were washed with aqueous sodium hydrogen carbonate and brine, dried with sodium sulfate, filtered, and evaporated in vacuo. The residue was purified by column chromatography (10 mL min^−1^) to give the desired product **13**.

#### 3.1.5. General Procedure for the Click Reaction

The conjugates **8**, **10**, **14** and **16** were synthesized using the following procedure: tralopyril terminal alkyne derivatives (compounds **7** or **13**, 1 mmol) and an azidoglycose (compounds **2** or **4**, 1 mmol) were dissolved in *t*-BuOH (3 mL). The reaction was initiated by the addition of CuSO_4_·5H_2_O (0.2 mmol) and sodium ascorbate (0.4 mmol) in distilled water (3 mL). The mixture was stirred at 60 °C until TLC indicated the disappearance of the starting materials. The mixture was poured into distilled water (20 mL), and the product was extracted three times with CH_2_Cl_2_ (20 mL). The organic layer was dried with Na_2_SO_4_ and filtered. The solvent was removed under reduced pressure. The residues were purified by column chromatography (1:2 petroleum ether/ethyl acetate) to obtain the glycosyl tralopyril conjugates **8**, **10**, **14** and **16**.

*4-Bromo-2-(4-chlorophenyl)-1-((1-(2,3,4,6-tetra-O-acetyl-beta-d-glucopyranosyl)-1H-1,2,3-triazol-4-yl)methyl)-5-(trifluoromethyl)-1H-pyrrole-3-carbonitrile* (**8**). ^1^H-NMR (500 MHz, DMSO-*d*_6_) δ 8.31 (s, 1H), 7.66–7.59 (m, 4H), 6.31 (d, *J* = 9.2 Hz, 1H), 5.62 (t, *J* = 9.4 Hz, 1H), 5.53 (t, *J* = 9.5 Hz, 1H), 5.28 (s, 2H), 5.17 (t, *J* = 9.8 Hz, 1H), 4.34 (m, 1H), 4.16–4.05 (m, 2H), 2.02 (s, 3H), 2.00 (s, 3H), 1.96 (s, 3H), 1.77 (s, 3H). ^13^C-NMR (126 MHz, DMSO-*d*_6_) δ 170.04, 169.59, 169.42, 168.42, 144.43, 142.72, 135.87, 131.80, 129.33, 125.50, 122.08, 113.69, 102.68, 97.92, 83.86, 73.24, 72.10, 70.13, 67.43, 61.59, 43.30, 20.52, 20.42, 20.26, 19.81. ESI-MS *m*/*z*: 761 [M + 1]^+^, 783 [M + Na]^+^.

*2-(4-((3-Bromo-5-(4-chlorophenyl)-4-cyano-2-(trifluoromethyl)-1H-pyrrol-1-yl)methyl)-1H-1,2,3-triazol-1-yl)-6-(methoxycarbonyl)tetrahydro-2H-pyran-3,4,5-triyl triacetate* (**10**). ^1^H-NMR (400 MHz, DMSO-*d*_6_) δ 8.31 (s, 1H), 7.62 (m, 4H), 6.30 (d, *J* = 9.1 Hz, 1H), 5.62 (t, *J* = 9.3 Hz, 1H), 5.52 (t, *J* = 9.5 Hz, 1H), 5.28 (s, 2H), 5.17 (t, *J* = 9.7 Hz, 1H), 4.33 (m, 1H), 3.65 (s, 3H), 2.02 (s, 3H), 2.00 (s, 3H), 1.96 (s, 3H). ^13^C-NMR (101 MHz, DMSO-*d*_6_) δ 170.07, 169.61, 169.44, 168.45, 144.44, 142.73, 135.89, 131.82, 129.34, 125.51, 122.10, 113.71, 83.87, 73.26, 72.12, 70.14, 67.44, 61.60, 52.10, 43.31, 20.55, 20.45, 20.29. ESI-MS *m*/*z*: 748 [M + 1]^+^, 770 [M + Na]^+^.

*4-Bromo-2-(4-chlorophenyl)-5-(trifluoromethyl)-1-((1-(2,3,4,6-tetra-O-acetyl-beta-d-glucopyranosyl-1H-1,2,3-triazol-4-yl)methoxy)methyl)-1H-pyrrole-3-carbonitrile* (**14**). ^1^H-NMR (400 MHz, DMSO-*d*_6_) δ 8.26 (s, 1H), 7.67 (d, *J* = 1.1 Hz, 4H), 6.33 (d, *J* = 8.7 Hz, 1H), 5.66–5.51 (m, 2H), 5.36 (m, 2H), 5.19 (t, *J* = 9.5 Hz, 1H), 4.42 (s, 2H), 4.37 (m, 1H), 4.18–4.03 (m, 2H), 2.03 (s, 3H), 2.00 (s, 3H), 1.97 (s, 3H), 1.76 (s, 3H). ^13^C-NMR (101 MHz, DMSO-*d*_6_) δ 170.08, 169.62, 169.44, 168.52, 144.53, 143.05, 135.90, 131.97, 129.35, 125.35, 123.29, 113.63, 98.01, 83.86, 75.44, 73.29, 72.12, 70.16, 67.53, 61.85, 61.16, 20.55, 20.44, 20.30, 19.90. ESI-MS *m*/*z*: 792 [M + 1]^+^, 814 [M + Na]^+^.

*2-(4-(((3-Bromo-5-(4-chlorophenyl)-4-cyano-2-(trifluoromethyl)-1H-pyrrol-1-yl)methoxy)methyl)-1H-1,2,3-triazol-1-yl)-6-(methoxycarbonyl)tetrahydro-2H-pyran-3,4,5-triyl triacetate* (**16**). ^1^H-NMR (500 MHz, DMSO-*d*_6_) δ 8.25 (s, 1H), 7.71–7.63 (m, 4H), 6.33 (d, *J* = 8.8 Hz, 1H), 5.65–5.50 (m, 2H), 5.40–5.32 (m, 2H), 5.19 (t, *J* = 9.6 Hz, 1H), 4.42 (s, 2H), 4.36 (m, 1H), 3.70 (s, 3H),2.03 (s, 3H), 2.00 (s, 3H), 1.97 (s, 3H).^13^C-NMR (126 MHz, DMSO-*d*_6_) δ 170.09, 169.63, 169.44, 168.53, 144.53, 143.06, 135.90, 131.97, 129.35, 125.36, 123.29, 113.63, 103.29, 98.02, 83.86, 75.43, 73.29, 72.12, 70.16, 67.53, 61.84, 61.16, 52.11, 20.54, 20.43, 20.29. ESI-MS *m*/*z*: 778 [M + 1]^+^, 800 [M + Na]^+^.

#### 3.1.6. General Procedure for the Removal of Acetyl Groups

The conjugates **8**, **10**, **14** or **16** (1 mmol) were added to a solution of sodium methoxide in dry methanol (0.05 M, 15 mL). The resulting solution was stirred for 30 min at room temperature. The mixture was neutralized with Amberlite IR120 (H^+^) resin and filtered. Subsequently, the filtrate was evaporated. The residues were purified by column chromatography (9:1 CH_2_Cl_2_/MeOH) to afford the glycosyl tralopyril conjugate **9**, **11**, **15** or **17**.

*4-Bromo-2-(4-chlorophenyl)-1-((1-(beta-d-glucopyranosyl)-1H-1,2,3-triazol-4-yl)methyl)-5-(trifluoromethyl)-1H-pyrrole-3-carbonitrile* (**9**). ^1^H-NMR (400 MHz, DMSO-*d*_6_): δ 7.94 (s, 1H), 7.62 (m, 4H), 5.45 (d, *J* = 9.4 Hz, 1H), 5.27 (s, 2H), 3.90–3.88 (m, 2H), 3.70–3.65 (m, 3H), 3.50–3.48 (m, 1H).^13^C-NMR (101 MHz, DMSO-*d*_6_) δ 144.4, 141.6, 135.7, 131.9, 129.3, 127.1, 125.6, 122.3, 121.1, 113.7, 102.5, 97.9, 87.5, 80.0, 76.9, 72.0, 70.0, 60.7, 43.2. ESI-MS *m*/*z*: 594 [M + 1]^+^, 617 [M + Na]^+^. 

*Methyl6-(4-((3-bromo-5-(4-chlorophenyl)-4-cyano-2-(trifluoromethyl)-1H-pyrrol-1-yl)methyl)-1H-1,2,3-triazol-1-yl)-3,4,5-trihydroxytetrahydro-2H-pyran-2-carboxylate* (**11**). ^1^H NMR (400 MHz, MeOD) δ 7.89 (s, 1H), 7.48 (m, 4H), 5.59 (d, *J* = 9.3 Hz, 1H), 5.34 (s, 2H), 4.11 (d, *J* = 9.8 Hz, 1H), 3.89 (t, *J* = 9.2 Hz, 1H), 3.74 (s, 3H), 3.66 (t, *J* = 9.5 Hz, 1H), 3.53 (t, *J* = 9.1 Hz, 1H). ^13^C-NMR (101 MHz, MeOD) δ 170.49, 146.03, 143.76, 138.11, 132.83, 130.51, 127.10, 123.53, 122.96, 121.11, 120.24, 114.26, 103.51, 101.12, 89.14, 79.07, 77.80, 73.30, 72.66, 53.02, 44.17. ESI-MS *m*/*z*: 622 [M + 1]^+^, 644 [M + Na]^+^.

*4-Bromo-2-(4-chlorophenyl)-5-(trifluoromethyl)-1-((1-(beta-d-glucopyranosyl-1H-1,2,3-triazol-4-yl)methoxy)methyl)-1H-pyrrole-3-carbonitrile* (**15**). ^1^H-NMR (400 MHz, DMSO-*d*_6_) δ 8.11 (s, 1H), 7.74–7.62 (m, 4H), 5.50 (d, *J* = 9.2 Hz, 1H), 5.40 (s, 2H), 5.38 (d, *J* = 5.9 Hz, 1H), 5.31 (d, *J* = 4.9 Hz, 1H), 5.18 (d, *J* = 5.5 Hz, 1H), 4.64 (t, *J* = 5.5 Hz, 1H), 4.39 (s, 2H), 3.69 (m, 2H). ^13^C-NMR (101 MHz, DMSO-*d*_6_) δ 144.55, 142.23, 135.97, 131.99, 129.45, 125.37, 123.54, 113.68, 98.01, 87.49, 80.01, 76.95, 75.58, 72.14, 69.59, 61.36, 60.80. ESI-MS *m*/*z*: 624 [M + 1]^+^, 646 [M + Na]^+^. 

*Methyl 6-(4-(((3-bromo-5-(4-chlorophenyl)-4-cyano-2-(trifluoromethyl)-1H-pyrrol-1-yl)methoxy)methyl)-1H-1,2,3-triazol-1-yl)-3,4,5-trihydroxytetrahydro-2H-pyran-2-carboxylate* (**17**). ^1^H-NMR (400 MHz, DMSO-*d*_6_) δ 8.18 (s, 1H), 7.70–7.57 (m, 4H), 5.71 (d, *J* = 9.3 Hz, 1H), 5.57 (m, 2H), 5.39 (s, 2H), 4.41 (s, 2H), 4.15 (d, *J* = 9.6 Hz, 1H), 3.82 (m, 1H), 3.66 (s, 3H). ^13^C-NMR (101 MHz, DMSO-*d*_6_) δ 168.78, 144.52, 142.39, 135.93, 131.91, 129.37, 125.31, 123.71, 113.66, 103.33, 87.07, 77.46, 76.10, 75.50, 71.61, 71.19, 61.25, 52.12. ESI-MS *m*/*z*: 652 [M + 1]^+^, 674 [M + Na]^+^.

#### 3.1.7. General Procedure for the Removal of Methyl Ester Groups

KOH (1.3mmol) was added to a solution of **11** or **17** (1 mmol) in MeOH (8 mL) at 0 °C. The reaction mixture was stirred overnight at room temperature and acidified via treatment with Amberlite IR 120H^+^. Filtration, evaporation under reduced pressure, and purification afforded **12** or **18** as a white solid.

*6-(4-((3-Bromo-5-(4-chlorophenyl)-4-cyano-2-(trifluoromethyl)-1H-pyrrol-1-yl)methyl)-1H-1,2,3-triazol-1-yl)-3,4,5-trihydroxytetrahydro-2H-pyran-2-carboxylic acid* (**12**). ^1^H-NMR (400 MHz, MeOD) δ 7.91 (s, 1H, Ar-H), 7.50 (q, *J* = 8.6 Hz, 4H, Ar-H), 5.60 (d, *J* = 9.3 Hz, 1H, H-1), 5.36 (s, 2H , NCH_2_), 4.07 (d, *J* = 9.7 Hz, 1H, H-5), 3.89 (t, *J* = 9.1 Hz, 1H, H-3), 3.67 (t, *J* = 9.4 Hz, 1H, H-4), 3.55 (t, *J* = 9.1 Hz, 1H, H-2). ^13^C-NMR (101 MHz, MeOD) δ 171.75, 146.05, 143.80, 138.13, 132.83, 130.51, 130.25, 127.09, 123.48, 122.97, 121.14, 120.29, 114.26, 103.47, 100.14, 89.16, 78.99, 77.96, 73.38, 72.70, 44.21. ESI-MS *m*/*z*: 608 [M + 1]^+^, 630 [M + Na]^+^.

*6-(4-(((3-Bromo-5-(4-chlorophenyl)-4-cyano-2-(trifluoromethyl)-1H-pyrrol-1-yl)methoxy)methyl)-1H-1,2,3-triazol-1-yl)-3,4,5-trihydroxytetrahydro-2H-pyran-2-carboxylic acid* (**18**). ^1^H-NMR (500 MHz, DMSO-*d*_6_) δ 8.17 (s, 1H), 7.71–7.60 (m, 4H), 5.65 (d, *J* = 9.3 Hz, 1H), 5.39 (s, 2H), 4.41 (m, 2H), 3.99 (d, *J* = 9.7 Hz, 1H), 3.79 (t, *J* = 9.1 Hz, 1H), 3.50 (t, *J* = 9.3 Hz, 1H), 3.43 (t, *J* = 8.9 Hz, 1H). ^13^C-NMR (126 MHz, DMSO-*d*_6_) δ 169.70, 144.54, 142.35, 135.96, 131.91, 129.39, 125.32, 123.72, 113.66, 103.31, 97.97, 87.14, 77.80, 76.32, 75.49, 71.70, 71.21, 61.26. ESI-MS *m*/*z*: 638 [M + 1]^+^, 660 [M + Na]^+^.

### 3.2. Phytotoxicity Assay to Tea

Seedlings of Fuding white tea were grown as previously described [14]. At eight weeks after sowing, average-sized seedlings were selected for the experiments. Four treatments were tested, namely, three concentrations of three conjugates and tralopyril (0.02, 0.2 and 2 mM), along with the untreated control (aqueous solution containing 0.1% Tween 80 and 10% methanol). A volume of 100 μL was applied to the whole tea shoots (a bud with two leaves) for each tea seedling. The score method used to assess the phytotoxic effect of the test substances was conducted according to previously described procedures [18,19]. Weekly observations of the phytotoxic symptoms for seven weeks in each plot and their corresponding scores were pooled and compared with the untreated control. Finally, the phytotoxic effect of the test substances was graded according to the previously described standard [18,19].

### 3.3. Sap Collection from Ricinus communis L. Seedlings and Analysis

Castor bean (*R. communis* L.) seeds were obtained from the Agricultural Science Academy of Zibo (Shandong, China), and grown as previously described [2]. At 6 days after sowing, average-sized seedlings were selected for the experiments. Phloem sap was collected from the upper part of the hypocotyl according to previously described methods [2,20,24]. The endosperm of seedlings was carefully removed without bending or crushing the cotyledons. These latter organs were incubated in buffer solution containing 20 mM MES (pH 5.5), 0.25 mM MgCl_2_, and 0.5 mM CaCl_2_ supplemented with 200 μM **9**, **11**, **12, 15**, **17** and **18** or 200 μM chlorfenapyr. After 1 h of incubation, the hypocotyl was severed in the hook region for phloem exudation, and the collected sap was stored in ice until analysis.The phloem sap was analyzed by HPLC (HP 1260 system, Agilent Technologies, Santa Clara, CA, USA, equipped with a quaternary pump, autosampler, and UV–visible detector) after dilution with pure water (phloem sap/pure water, 1/3, *v*/*v*). Separations were performed with a C_18_ reversed-phase column (5 μm, 150 × 4.6 mm i.d., Agilent Technologies) at 25 °C. The mobile solvent system consisted of acetonitrile and water containing 0.1% trifluoroacetic acid (40/60, *v*/*v* for **9**, **11**, and **12**; 45/55, *v*/*v* for **15**, **17**, and **18**) or water (80/20, *v*/*v* for chlorfenapyr) at a flow rate of 0.5 mL min^−1^. The detector wave length was 210 nm for **9**, **11**, **12, 15**, **17**, **18** and chlorfenapyr. External calibration was used to determine their concentrations. A series of standard solutions (0.5, 1, 5, 10, 25 and 50 μM) for the calibration curves was prepared in methanol. Their linear equations of the different compounds are shown in Table 6.

### 3.4. Insecticidal Activity of Six Conjugates Against P. xylostella

Third-instar larvae of *P. xylostella* and its artificial fodder were obtained from the He Nan Ji Yuan Bai Yun Industrial Co., Ltd. (Jiyuan, Henan, China). The bioactivities of **9**, **11**, **12**, **15**, **17**, **18** and chlorfenapyr on the third-instar larvae of *P. xylostella* were assessed by mixing them directly into the fodder. Three concentrations (1, 2and 4 mM) of the six conjugates and chlorfenapyr were dissolved in methanol and acetone (10/90, *v*/*v*). Each test solution (1 mL) and artificial fodder (1 g) were placed into Petri dishes (9 cm in diameter). Artificial fodder (1 g) containing methanol and acetone (1 mL, 10/90, *v*/*v*), but not the tested compound, was used as the control. At 8 h after the solvent was evaporated, ten *P. xylostella* larvae were then introduced into each dish. The dishes were incubated at 26 °C and 70% relative humidity under a photoperiod of 16:8 h (light/dark). All treatments were repeated four times. Mortalities were determined at 48 h and 72 h post treatment.

### 3.5. Systemic Mobility on Cabbage and Systemic Insecticidal Activity of Conjugates Against P. xylostella

Two-weeks-old seedlings of *Brassica oleracea* were fertilized with half-strength Hoagland’s solution once a week. The growing plants were used in the tests for three weeks. The roots of the cabbage seedlings were dipped in the conjugates (4 mM) and chlorfenapyr aqueous solution (50 mL) containing Tween80 (0.1%) and methanol (5%). The aqueous solution containing Tween80 (0.1%) and methanol (5%) was used as the control. All treatments were repeated three times. The leaves were harvested at 48 h and 72 h after the root-applied chemical and then were divided into two parts. One part of the harvested samples was stored at −15 °C until they could be analyzed. Another part of the harvested samples was used to the assessments of systemic insecticidal activity.

#### 3.5.1. Analysis

Plant samples (1 g) were ground into powder, transferred to volumetric flasks, and ultrasonically treated for 15 min with 10 mL of methanol. The extracts were filtered, and filtrate residues were reextracted twice. The combined extracts were rotary-evaporated to dryness at 65 °C, and the residues were transferred in 2 mL of methanol to a solid-phase extraction (SPE) cartridge (AccuBond C18 SPE, Agilent Technologies). The SPE columns were eluted in another 2 mL of methanol, and the collected solution was evaporated to a volume of 1 mL. The resulting solutions were diluted with methanol and used for HPLC analysis. The HPLC analyses were performed according to above mentioned procedures. The recovery rate ranged from 85% to 96%, which was acceptable for the test.

#### 3.5.2. Assessments of Systemic Insecticidal Activity

The harvested fresh leaves were placed into Petri dishes (9 cm in diameter). Ten *P. xylostella* larvae were then introduced into each dish. The Petri dishes were incubated at 26 °C and 70% relative humidity under a photoperiod of 16:8 h (light/dark). All treatments were repeated three times. Mortalities were determined at 48 h and 72 h post treatment.

### 3.6. Physicochemical Properties

The physicochemical properties (molecular mass [MW], the octanol : water partition coefficient [logD], acid dissociation constant polar [pKa], number of hydrogen bond donors [HBDs], and number of hydrogen bond acceptors [HBAs]) of the six conjugates, tralopyril and chlorfenapyr were predicted using ACD LogD suite version 12.01 software (Advanced Chemistry Development, Inc., Toronto, ON, Canada).

## 4. Conclusions

In summary, six new conjugates containing tralopyril and pyranose moieties (glucose, methyl glucuronate or glucuronic acid) were synthesized via click chemistry. The applied mild reaction conditions provided a application case for the addition of a sugar moiety to pesticides. No phytotoxicity symptoms were observed up to 63 days after smearing 2 mM of the six conjugates on seedlings, which indicated that the introduction of a sugar group may solve the difficult phytotoxicity problem of tralopyril. The phloem mobility tests on *Ricinus* system demonstrated that the presence of a glucuronic acid core conferred more phloem mobility to tralopyril than glucose. The translocation of conjugates **12** and **18** with a glucuronic acid core may involve two components of carrier-mediated systems and an ion trap mechanism. This inference requires further confirmation. Insecticidal activity results indicated that the methoxymethyl group on the pyrrole N of tralopyril moiety was a determinant factor in the insecticidal activity, and the pyranose structure also affects the insecticidal activity. Their insecticidal mechanism of action should be confirmed by obtaining direct evidence in further experiments. Based on the systemic mobility and systemic insecticidal activity test results, conjugate **18** with a glucuronic acid group and methoxymethyl group on the pyrrole N of tralopyril could be a promising systemic pro-insecticide.

## Figures and Tables

**Table 1:** Phytotoxicity of tralopyril, **9**, **11**, **12**,**15**, **17** and 1**8** to tea shoots.

Phytotoxicity Symptom Scales *^a^*		Tralopyril			9	11	12	15	17	18
Symptoms	Score (%)	0.02 mM	0.2 mM	2 mM	2 mM	2 mM	2 mM	2 mM	2 mM	2 mM
Injury to leaf tips	20	0	20	20	0	0	0	0	0	0
Injury to leaf surfaces	20	0	20	20	0	0	0	0	0	0
Leaf wilting	20	0	20	20	0	0	0	0	0	0
Necrosis	10	0	10	10	0	0	0	0	0	0
Vein clearing	10	0	0	10	0	0	0	0	0	0
Epinasty	10	0	0	0	0	0	0	0	0	0
Hyponasty	10	0	0	0	0	0	0	0	0	0
Total score		0	70	80	0	0	0	0	0	0
grade *^b^*		1	7	8	1	1	1	1	1	1

*^a^* Phytotoxicity symptoms and their scores were determined according to references [18,19]. *^b^* The phytoxicity effect was graded as per the following scale: 0–10, 1; 11–20, 2; 21–30, 3; 31–40, 4; 41–50, 5; 51–60, 6; 61–70, 7; 71–80, 8; 81–90, 9; 91–100, 10.

**Table 2:** Predicted physicochemical descriptor MW, log D, pKa, HBD, HBA of tralopyril, chlorfenapyr, **9**, **11**, **12**, **15**, **17** and 1**8** *^a^*.

Feature	MW (D)	Log D (pH 5.0)	Log D (pH 8.0)	pKa	HBD	HBA	Log P	Log Cf *^c^*
Tralopyril	349.53	4.75	4.75	14 *^b^*	1	2	4.75	<−99
chlorfenapyr	407.61	4.76	4.76	14	0	3	4.76	<−99
**9**	592.75	2.02	2.02	14	4	10	2.02	<−99
**11**	620.76	2.50	2.50	14	3	11	2.50	<−99
**12**	606.73	0.48	−1.84	2.75	4	11	2.15	0.32
**15**	622.78	2.29	2.29	14	4	11	2.29	<−99
**17**	650.79	2.49	2.49	14	3	12	2.49	<−99
**18**	636.76	0.63	−1.69	2.77	4	12	2.30	0.49

*^a^* All parameters were computed using ACD/Lab sversion 12.01 software. *^b^* Assumed nonacidic and assigned pKa of 14. *^c^* Log Cf values were calculated for a short plant [6,26]. Plant parameters for these calculations are L = 0.15 m, l = 0.05 m, r = 5 × 10^−6^ m, v = 3.0 × 10^−4^ m s^−1^, pH (phloem) = 8, and pH (apoplast) = 6. The permeability of the membrane was calculated using log P = 1.2 (log Kow) − 7.5.

**Table 3:** The fatality rates of tralopyril, **9**, **11**, **12**, **15**, **17** and 1**8** against *P. xylostella.*

	48 h Fatality Rate (%)			72 h Fatality Rate (%)		
	1 mM	2 mM	4 mM	1 mM	2 mM	4 mM
Chlorfenapyr	100.00	100.00	100.00	100.00	100.00	100.00
**9**	0.00	0.00	0.00	0.00	0.00	0.00
**11**	0.00	0.00	0.00	0.00	0.00	0.00
**12**	0.00	0.00	0.00	0.00	0.00	0.00
**15**	0.00	10.00	100.00	10.00	30.00	100.00
**17**	100.00	100.00	100.00	100.00	100.00	100.00
**18**	0.00	33.33	93.33	0.00	96.66	100.00

**Table 4:** Detected concentration for the harvested leaves from the treatment of root-applied compound.

	Root-Applied Compound	Concentration of Detected Compound (µg/g)			
		15	17	18	Chlorfenapyr
48 h harvested leaves	**15**	252.96	-	-	-
	**17**	-	ND *^a^*	34.98	-
	**18**	-	-	1905.27	-
	Chlorfenapyr	-	-	-	ND
72 h harvested leaves	**15**	47.68	-	-	-
	**17**	-	ND	68.79	-
	**18**	-	-	1947.95	-
	Chlorfenapyr	-	-	-	ND

*^a^* ND = not detected.

**Table 5:** The systemicinsecticidal activity of the conjugates against *P. xylostella*.

	Root-Applied Compound	48 h Mortalities Post Feeding (%)	72 h Mortalities Post Feeding (%)
48 h harvested leaves	**15**	0.00	36.66
	**17**	0.00	10.00
	**18**	20.00	93.33
	Chlorfenapyr	0.00	0.00
	CK *^a^*	0.00	0.00
72 h harvested leaves	**15**	0.00	0.00
	**17**	0.00	46.66
	**18**	53.33	100.00
	Chlorfenapyr	0.00	0.00
	CK	0.00	0.00

*^a^* CK= control.

**Table 6:** The linear equations of the compounds.

Compound	Linear Equations	R^2^
Chlorfenapyr	y = 38.04x + 68.58	0.9974
**9**	y = 25.96x + 8.59	0.9998
**11**	y = 28.50x + 9.55	0.9999
**12**	y = 29.91x + 9.69	0.9998
**15**	y = 50.09x – 34.16	0.9987
**17**	y = 33.77x – 10.19	0.9968
**18**	y = 35.45x + 11.11	0.9995
